# Genetic predisposition to chikungunya – a blood group study in chikungunya affected families

**DOI:** 10.1186/1743-422X-6-77

**Published:** 2009-06-16

**Authors:** Lokireddy Sudarsanareddy, Vemula Sarojamma, Vadde Ramakrishna

**Affiliations:** 1 Department of Biotechnology, Sri Krishnadevaraya University, Anantapur – 515 003, India; 2 Department of Community Medicine, Government Medical College, Anantapur – 518001. India

## Abstract

Chikungunya fever is a viral disease transmitted to humans by the bite of CHIKV virus infected *Aedes *mosquitoes. During monsoon outbreak of chikungunya fever, we carried out the genetic predisposition to chikungunya in disease affected 100 families by doing blood group (ABO) tests by focusing on individuals who were likely to have a risk of chikungunya and identified the blood group involved in susceptibility/resistance to chikungunya. In the present study, based on blood group antigens, the individuals were kept in four groups – A (108), B (98), AB (20) and O (243). The result obtained was showed all Rh positive blood group individuals are susceptible to chikungunya fever. Among ABO group, the blood group O +ve individuals are more susceptible to chikungunya than other blood groups. No blood group with Rh negative was affected with chikungunya, it indicates Rh -ve more resistance to chikungunya.

## 

Chikungunya, an arboviral disease transmitted by *Aedes *mosquitoes, has recently increased dramatically in incidence and geographic extent. Large outbreaks have affected islands of the Indian Ocean, India and other parts of South and Southeast Asia, Africa and most recently Italy [1-3]. Chikungunya virus is an important human pathogen, a member of the Alphavirus genus in the family Togaviridae that causes a syndrome characterized by fever, chills, headache and severe joint pain with or without swelling (usually the smaller joints). The name is derived from the Makonde word meaning 'that which bends up' in reference to the stooped posture developed as a result of the arthritic symptoms of the disease [4,5]. The outbreak was first investigated in February 2006 in Andhra Pradesh and then in March 2006 in Karnataka by health officials of the country who confirmed the occurrence of chikungunya virus in the region [6,7]. The outbreak in India started in the end of 2005 and has an attack rate of 4–45% [8]. No treatment or vaccine is available, and relatively little research has been conducted into pathogenesis of chikungunya, compared with that of other arboviruses, such as dengue.

Genetic factors are important in the predisposition to various diseases. Complex diseases are generally influenced by more than one gene or environmental factor, and as a consequence, do not exhibit a simple mode of inheritance. In community, although only a small percentage of exposed individuals will develop the disease. Some individuals often show variation in susceptibility/resistance to certain diseases. Therefore, host susceptibility, genetic factors and, possibly environmental factors may be important for the development of diseases. During July – October 2006 year monsoon outbreak of chikungunya fever in Southern parts of India particularly in Anantapur District of Andhra Pradesh, has led us to carryout the genetic predisposition of chikungunya in affected families to identify susceptible or resistant blood group by analyzing the blood group in the chikungunya affected people. This type of work has not been carried out by any scientific group. In the present study, we designed to focus on individuals who were likely to have a risk of chikungunya and identified the blood group involved in susceptibility/resistance to chikungunya in the chikungunya affected families.

During outbreak of chikungunya in Andhra Pradesh, India, a total of 100 chikungunya affected families from nearby villages of Sri Krishnadevaraya University, with on and average of 5 members (age of 10 – 70 years) in each family were selected based on disease symptoms for this study. Blood samples were collected from each subject with their prior written consent and identified Blood groups by using commercial blood group kit containing Anti -A, Anti-B and Anti-D monoclonal antibody reagents. Statistical analysis was performed by using GraphPad InStat software.

Among 468 individuals of 100 families surveyed, 95 subjects (20%) reported no chikungunya fever and remaining 80% suffering with all chikungunya symptoms with fever, headache, and severe joint pains. Based on blood group Antigens, the individuals were kept in four groups – A (108), B (98), AB (20) and O (243) (figure 1). Among A blood group, 98% people (106) are Rh positive and 80% (85) are suffering with chikungunya. However in the blood group B, 60% are suffering from total of 94 Rh positive. In the case of AB group, all 20 individuals are Rh positive but only 5 subjects shown chikungunya. Nearly 52% of total study group was shown O blood group. Among 243 subjects, 235 were Rh positive and 96% (227) of Rh +ve cases were shown chikungunya symptoms. No Rh negative individual of any blood group showing any type of chikungunya fever symptoms (figure 2). In the individual familial studies, we also observed that the family who is having O positive blood group is completely affected with chikungunya fever. However, within the same family the person having O negative is not showing any symptoms of chikungunya.

**Figure 1:**
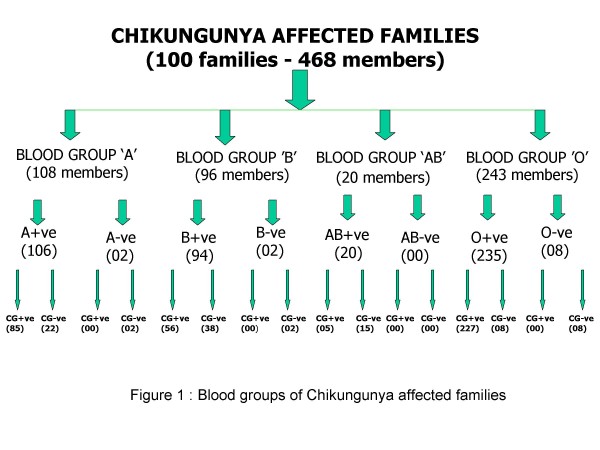
**Blood groups of chikungunya affected families**.

**Figure 2:**
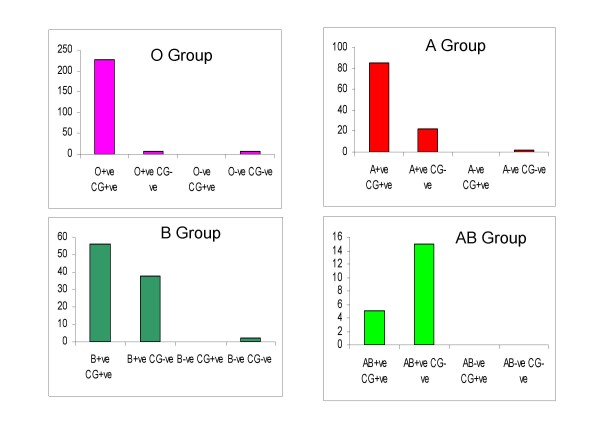
**Prevalence of chikungunya in blood groups**.

In conclusion, the present results state that all Rh positive blood group individuals are susceptible. Among them, the blood group O +ve individuals are more susceptible to chikungunya than other blood groups. No blood group with Rh negative was affected with chikungunya; it indicates Rh -ve more resistance to chikungunya.

## Competing interests

The authors declare that they have no competing interests.

## Authors' contributions

LS conducted the experiments with the patients' blood. LS also performed the final statistical analysis of the data and contributed to writing the paper. VS (she is a specialist in infectious diseases) gave us guidance in selecting the chikungunya patients for research during time of blood collection. VR supervised the overall project, designed experiments, analyzed the data and wrote the paper. All authors read and approved the final manuscript.
